# Cadmium Removal from Aqueous Solutions by Strain of *Pantoea agglomerans* UCP1320 Isolated from Laundry Effluent

**DOI:** 10.2174/1874285801812010297

**Published:** 2018-08-31

**Authors:** Leonila. M. L. Acioly, Davi Cavalcanti, Marcos C. Luna, José C. V. Júnior, Rosileide F. S. Andrade, Thayse A. de Lima e Silva, Camilo E. La Rotta, Galba M. Campos-Takaki

**Affiliations:** 1 Post Graduate Program in Biological Sciences, Federal University of Pernambuco, 50670-420, Recife, PE, Brazil; 2 Northeast Network for Biotechnology-RENORBIO, Federal Rural University of Pernambuco, 52171-900 Recife-PE, Brazil; 3 Autarchy of Higher Education of Garanhuns (AESGA), 55295-380 Garanhuns, Pernambuco, Brazil; 4 National Post-Doctorate Program (PNPD)-CAPES, Catholic University of Pernambuco, 50050-900, Recife, Pernambuco, Brazil; 5 Nucleus of Research in Environmental Sciences and Biotechnology, Catholic University of Pernambuco, 50050-590, Recife, PE, Brazil

**Keywords:** *Pantoea agglomerans*, Pollution, Heavy metal, Biosorption, Absorption isotherms, Pollutants

## Abstract

**Background:**

Cadmium (Cd), which is a deadly heavy metal of work-related and environmental concern, has been recognized as a substance that is teratogenic and carcinogenic for humans. Therefore, the need to develop low-cost adsorbents to remove heavy metals from aqueous solution has greatly increased. Adsorbents such as *Pantoea agglomerans* biomass have been used.

**Aims:**

We investigated the biotechnological potential of *Pantoea agglomerans* for the biosorption of cadmium from aqueous solution.

**Patients and Methods:**

*Pantoea agglomerans* UCP1320 isolated from the effluent of a laundry industry was used to remove cadmium from aqueous solutions. Two approaches were compared using active or thermally inactivated biomass. Three different cadmium concentrations of 1, 10 and 100 ppm were used under constant stirring at temperatures of 25°C and 35°C as was pH of 3.0, 5.0 and 7.0. Variable incubation times of 1, 6, and 24h were also studied.

**Results:**

The results showed that the temperature did not influence the uptake of metal by living cells nor by inactive bacterial biomass. However, increasing the pH had a positive effect on removing intermediate concentrations of cadmium. Low concentrations of cadmium were completely removed by both live and inactive biomass.

**Conclusion:**

*Pantoea agglomerans* biomass was shown to have a promising performance for the biotechnological removal of cadmium which had been dissolved in aqueous solution.

## 1 INTRODUCTION

The constant discharge of different pollutants such as organic compounds and heavy metals into the environment is causing growing concern throughout the entire world. Several human activities have driven the increase in heavy metals being discharged into the environment. The question now is how to combat the rise in concentrations since these jeopardize human and animal health. In parallel, scientific advances have dealt with how to exploit natural resources without provoking the detrimental effects caused by many pollutants that have provoked environmental problems over many years [1-3]. In this context, the use of bioremediation as a tool offers the possibility of removing or transforming these long-studied pollutants into harmless compounds, based on the natural activity of various microorganisms. In addition, bioremediation has been gaining public attention and acceptance when it can be shown that this can be done at low cost, is effective, and it is feasible to apply it *in locus*. Compared to other non-biological technologies, bioremediation is one of the most promising and least costly alternatives for removing pollutants from soil, air and water [4-6].

The presence of heavy metals such as cadmium, chromium, lead, zinc, copper and mercury are a constant threat to the environment since these metals tend to accumulate, thereby adversely affecting several biological niches [7, 8].

Cadmium is a heavy metal that is difficult to degrade. It is known for its high binding capacity to biomolecules and especially to the enzymes present in the respiratory chain, thus leading to oxidative stress and physiological problems, including cancer [8, 9]. Most cadmium is obtained as a by-product when smelting zinc, lead, or copper ores.

Metal (Cadmium) by-products are mostly used in metal plating and to make pigments, batteries, and plastics. This metal can be discharged into the soil in the form of urban, industrial sludge and in wastewaters. In addition, it can also be easily absorbed by and translocated in plants which are how it enters the food chain of humans and cattle. The results of some animal studies show that animals given cadmium-contaminated food and water develop high blood pressure, iron-poor blood, liver disease, nerve damage or brain damage [10, 11].

Conventional methods of removing metals such as precipitation, oxidation or reduction have been commonly used to remove heavy metal from industrial wastewater. They are ineffective or expensive, however. Substitute methods of metal removal and revival based on biological resources have been considered. Certain types of microbial biomass can retain comparatively high quantities of metal by means of passive processes known as biosorption [12, 13]. Such processes are of industrial interest, since the removal of potentially hazardous concentrations of cadmium can be achieved [14, 15].

Microorganisms use mainly two different processes for transforming and immobilizing heavy metals. One is called bioaccumulation, which is based on incorporating the metal pollutant into the living biomass. In the other main process, called biosorption, metallic ions remain on the cellular surface by different mechanisms [16]. Both methods show variable advantages related to operational costs, the volume of the residue to be treated and the biological waste to be disposed of, the efficiency of detoxification and removal, nutritional requirements and dilutions. However, the biological removal process is quite efficient, fast and is performed under mild operational conditions [17].

In the aquaculture industry is currently controlled with copper as a biocide paints problem. The development of these activities has resulted in marine and freshwater sediments next to the culture centers high levels of copper. In this sense, *Pantoea agglomerans* showed a high resistance for copper, as well as ability to remove copper. The aquaculture industry paints floating cages to prevent microorganisms adhering to them but this leads to high levels of copper in the paint being in contact with marine and freshwater sediments. *Pantoea agglomerans* has been used as it has a high resistance to copper and is able to remove copper from these sediments. The authors suggest that marine bacteria could be used in a biological system to remove copper [18]. This article describes the characterization of the biotechnological potential of the strain of *Pantoea agglomerans* on cadmium in aqueous solutions when biosorption and bioaccumulation bioprocesses are used.

## 2 MATERIAL AND METHODS

### 2.1 Microorganism, Culture Media and Conditions

*Pantoea agglomerans* is a Gram-negative bacterium that belongs to the family Enterobacteriaceae. The bacterium was isolated from laundry effluent and was identified as *Pantoea agglomerans* (UCP 1320) according to Acioly *et al*. [19] and deposited in the Culture Collection UCP (Universidade Católica de Pernambuco), Recife-PE, Brazil, and was registered in the WFCC (World Federation for Culture Collection).

The biomass from *P. agglomerans* was obtained following the methodology described by Hernandez *et al*. [20]. The bacterium was cultivated overnight in metal-free Luria-Bertani solid medium agar [21] at 30°C. Then colonies were harvested and suspended in normal saline solution, centrifuged at 10.000*g* for 20 min at 10°C, and washed twice with the same solution and finally lyophilized. To obtain the inactivated biomass, the cell suspensions were submitted to autoclaving at 121°C for 15 min [22]. After cooling, cells were lyophilized and kept in a desiccator until constant weight.

### 2.2 Experiments of Biosorption and Bioaccumulation

These experiments were performed using 100 mg of active or inactivated biomass per each 250 mL flask filled with 100 mL cadmium chloride solutions of 1.0, 10.0 and 100.0 ppm of (Cd^2+^) in normal saline solution. The effect of pH was evaluated by adjusting the pH with HCl or NaOH (1 mol.L^-1^) to final values of 3.0, 5.0 and 7.0. Finally, the effect of different temperatures was evaluated at 25 and 35°C. All assays were incubated for 24h under continuous stirring at 150 rpm. Samples of (5mL) at T_0_, T_1_, T_6_ and T_24_ h were taken. All assays were performed in duplicate and also two samples were taken each time.

### 2.3 Electrochemical Method for Quantifying Cadmium

Each sample was centrifuged at 9.000 rpm for 20 min, and the cell free supernatants were used to quantify cadmium. Residual Cd (II) was analyzed by Anodic Stripping Voltammetry (ASV) and Square wave Voltammetry (SWV). Stripping analysis is an analytical technique that involves the pre-concentration of a metal phase [23-25]. A glassy carbon electrode (0.5 cm in diameter) was used as a working electrode; a Platinum wire as a counter electrode and an electrode of AgCl in saturated KCl was used as a reference electrode. As support electrolytes, 100 mmol L^-1^ solutions of H_3_PO_4_ or KCl were used. Quantification was performed from calibration curves of variable concentrations of Cd(II) as CdCl_2_ in 20 mmol L^-1^ HCl ranging from 1.0 to 20 ppm. Electrochemical parameters for ASV include: E_c_ = 0.6 V, T_c_ = 60 s, E_d_ = - 1.4 V and t_d_ = 60 s. And for SWV of: F = 25Hz; E_0_ = -1.4 V, E_f_ = 0,0, E_step_ = 0.005 and wave width = 0.025 mV.

### 2.4 Determining Removal and Calculating Absorption Isotherms

Removal percentages were determined as per Equation **1**:

(1) $$\mathrm{Removal},\%=(C_{o}-C_{f}/C_{0})\text{∗}100$$

where C_o_ corresponds to the initial concentration of Cd(II) and C_f_, the final concentration after the sorption processes caused by the chitosan membrane. The metal uptake capacity, *q*, for a Cd(II) ion was calculated as:

(2) $$q_{t}=V/m(C_{0}-C_{f})$$

where *q_t_, C_0_, C_f_, V* and *m* are the amount of solute adsorbed per unit weight of adsorbent (mg g^-1^) at t (min), the initial metal ion concentration, the final metal ion concentration (mg L^-1^), the volume (L) of the solution and the dry weight of adsorbent (g), respectively. The amount of cadmium adsorbed by adsorbents, *q*, was determined by:

(3) $$q_{e}=V/m(C_{0}-C_{e})$$

In this Equation, *q_e_* and *C_e_* are the amount of solute adsorbed per unit weight of adsorbent (mg/g) at equilibrium and the equilibrium concentration of cadmium (mg/L), respectively. The three most widely used adsorption isotherms are the Langmuir, Freundlich and Dubinin-Radushkevich (D–R) isotherms. In this study, experimental data were analyzed in terms of the Langmuir adsorption isotherm equation in linear form which is:

(4) $$1/q_{e}=(1/q_{\max.K_{L}}).1/C_{e}+1/q_{\max}$$

where *q*_max_ is the maximum adsorption capacity of adsorbent (mg/g) and *K_L_* is the Langmuir constant related to the energy of adsorption (L mg^-1^). *K_L_* and *q*_max_ can be calculated from the slope and intercept of the linear plot of 1/*q_e_* *versus* 1/*C_e_*.

## 3 RESULTS

### 3.1 Effect of pH, Temperature and Incubation Time Over Cadmium Biosorption By Biomass of *P. agglomerans*

As a control experiment, we used thermal inactivated cells of *P. agglomerans* in an attempt to determine if the removal was caused by the biomass itself (sorption processes) or by any other metabolic process involved (bioremediation processes). When the inactivated cells were put in contact with low concentrations of cadmium, very high to almost complete removal was achieved, with some interesting exceptions.

Fig. (**1**) shows the results in terms of the Cd (II) removal achieved with the inactivated cells of *P. agglomerans*, when incubated at temperatures of 25°C (A) or 35°C (B), respectively. As can be seen, noticeable increases were observed at 25°C, when the biomasses were incubated at higher pH values. This observation was even more evident when pH changed from 3.0 to 5.0, while between 5.0 and 7.0 the increase was almost insignificant.

On the other hand, when the incubation period was extended up to 24h, the highest removal of Cd (II) was achieved in pH values between 5.0 and 7.0. This amounted to the removal of 100% at 24h. Conversely, at a higher temperature of 35°C maximum removals of 100% were achieved after 6h of incubation.

The Fig. (**2**) shows the effect of the pH on cadmium removals obtained using living cells of *P. agglomerans,* incubated at 25 or 35°C in aqueous solutions containing 10 ppm of Cd(II).

As can be observed, higher removal was achieved at lower temperatures and lower pH values. As such, the best overall results were obtained when *P. agglomerans* cells were incubated at pH 3.0 and 25°C. Interestingly, at the same incubation temperature, but with a pH of 7.0, a decrease of almost 20% in Cd (II) removal was observed, while at 35°C slight increases were observed at the same pH value. As we expected, the best results were achieved in all cases at higher incubation times. Thus, 100% of Cd (II) was removed from the aqueous solutions by the living cells after 24h, no matter the pH value or incubation temperature.

Table **1** summarizes the results obtained for the maximum removals achieved using 10 ppm of Cd (II) and both active and inactivated cells of *P. agglomerans.* When living cells of *P. agglomerans* were used both removal processes are expected to occur simultaneously, one related to the cell metabolism and the other to physical interactions between the heavy metals and the biological structures existing in the cells.

The global removal was slightly higher than the one observed for the sorption process due only to physic-chemical interactions, especially when the processes were performed at the higher temperature of 35°C. The best overall results were obtained at pH 6.0 and 35°C. Since these conditions are closely related to the physiological conditions where the bacterium grows, the increase in the removal can be expected to be related to the biological activity. When the maximum removals observed by the inactivated cells were subtracted from those obtained for the living cells, real removal values were obtained corresponding only to the cellular metabolism. Therefore, a maximum removal of 15% was observed for bacterial metabolism at pH 5.0 and 35°C. Since the values for removal observed for all pH values tested at 25°C were close to each other, this clearly indicates that the removal was mostly driven by the increase in the temperature rather than being due to pH at low concentrations of Cd (II).

### 3.2 Effect of Cd(II) Concentration Over Biosorption

The effect of Cd (II) concentration was tested only up to 100 ppm since higher concentrations of these heavy metals are rarely found above this limit in waste-waters.

As can be seen in Fig. (**3**), the increase of the Cd (II) removal occurs when the incubation time is extended, and the temperature and pH were increased. No significant variations were observed between pH values at 25 or 35°C. However, 10% increases in variation were observed between 1h and 6h of incubation as was a 10% increase in removal when inactivated cells were incubated for up to 24h at 25°C. Lower increases in removal of a maximum of 10% were observed when cells were incubated at 35°C for up to 6h and only 4% when incubated under the same conditions for up to 24h.

In general, the best results were achieved at pH 7.0 and 35°C after 24h. In contrast, Fig. (**4**) **s**hows the results obtained for removing the same concentration of Cd (II) by active cells of *P. agglomerans*. As can be observed, when we increase the concentration of Cd(II) by one log, removal decreases to one half of the value observed at lower concentrations of this metal. In both Fig. (**4A**, **B**) it can be observed that the best results were achieved at the lower incubation time of 1 h, and a slight increase in removal accompanied the increase in pH in all cases.

For higher concentrations of Cd (II) no relationship can be established between temperature changes since no significant variations were observed at 25 or 35 °C. However, what remains obvious is the fact that at longer incubation times, the probability of desorption can be expected between 6h and 24h, since lower concentrations of Cd (II) were removed from the solutions. This can imply that the metal exerts toxicity on the cells, when they are able to remove up to 70 ppm of cadmium, during the first hour, but then the metal caused cellular death and consequently for cellular lysis and this fosters releasing the metal releasing back to the solution.

### 3.3 Effect of Cd(II) Concentration Over Biosorption

The effect of Cd (II) concentration was tested only up to 100 ppm since higher concentrations of these heavy metals are rarely found above this limit in waste-waters. As can be seen in Fig. (**3**), an increase in removing Cd (II) followed when the incubation period was extended, and also when both the temperature and pH were increased when inactivated cells were used as sorbent material. No significant variations were observed between pH values at 25°C or 35°C. However, 10% increases in variation were observed between 1h and 6h of incubation as was a 10% increase in removal when inactivated cells were incubated for up to 24h at 25°C. Lower increases in removing a maximum 10% were observed when cells were incubated at 35°C for up to 6h and the increase was only 4% when incubated under the same conditions for up to 24h.

In general, the best results were achieved at pH 7.0 and 35°C after 24h. In contrast, Fig. (**4**) **s**hows the results obtained for removing the same concentration of Cd (II) by active cells of *P. agglomerans*. As can be observed, when we increase the concentration of Cd(II) by one log, removal decreases to one half of the value observed at lower concentrations of this metal. In both Fig. (**4A**, **B**) it can be observed that the best results were achieved at the lower incubation time of 1 h, and a slight increase in removal accompanied the increase in pH in all cases. For higher concentrations of Cd (II) no relationship can be established between temperature changes since no significant variations were observed at 25°C or 35°C. However, what remains obvious is the fact that at longer incubation times, the probability of desorption can be expected between 6h and 24h, since lower concentrations of Cd (II) were removed from the solutions. This can imply that the metal exerts toxicity on the cells, when they are able to remove up to 70 ppm of cadmium, during the first hour, but then the metal caused cellular death and consequently for cellular lysis and fosters releasing the metal releasing back to the solution.

Conversely, as to the observations made at low Cd (II) concentrations, the best results were achieved only at longer incubation times. Table **2** shows the summarized results obtained at these times for the incubation conditions used for both inactivated and active cells of *P. agglomerans*. As can be seen, no significant differences were observed between maximum Cd (II) removal values when both sorption and metabolic removals corresponding to the living cells were evaluated. However, lower results were obtained for the sorptive processes due only to the inactivated biomass. As such, the average value of removal caused only by the metabolism of the living cells was close to 6.00%.

Table **3** shows the removal rates calculated for each experiment during 24h of incubation. It was observed that the overall highest rates of 6.032 and 6.638 ppm h^-1^ were achieved at pH 7.0, 35°C and 100 ppm of Cd (II) for inactivated biomass and living cells, respectively. It must be noted that living cells of *P. agglomerans* also demonstrated good removal rates at pH 5.0 and 3.0 from low Cd (II) concentrations of 10 ppm.

In our study, the cadmium biosorption by *Pantoea agglomerans* showed a positive effect of removal using pH of between 5.0 - 6.0 and a temperature of 25 °C. The strain of *P. agglomerans* showed excellent removal capacity for Cd (II) at low concentrations of 1 ppm, in which both active and inactivated biomass were successful (data not shown).

## 4 DISCUSSION

The properties of cell wall constituents, such as peptidoglycan and the role of functional groups, such as carboxyl, amine and phosphonate are related to their biosorption potential [21]. The cell wall is the first component of the bacterial physiology that comes into contact with metallic ions, and this is where the solutes can be deposited either over its surface or inside it [25, 26]. Since the absorption of solutes by inactive cells is mostly extracellular, the chemical moieties present on the cell wall perform a key role during biosorption. In addition, the increase in pH promotes the formation of negative-charged functional groups by deprotonation which favors the electrostatic attraction and adsorption of cations [27]. This fact was clearly demonstrated when both active and inactivated cells of *P. agglomerans* were incubated at variable pH values.

The efficiency of bioaccumulation by living cells depends on the conditions under which they grow, their physiological state and their age. For example, Mapolelo and Torto [28] mentioned that a pretreatment of *S. cereviseae* cells with glucose 10 to 20 mmolL^-1^ enhanced removal of between 30 to 40% for Cd^2+^, Cr^3+^, Cu^2+^, Pb^2+^ and Zn^2+^. This observation was also reported by Stoll and Duncan [29] who studied the absorption of Cu^2+^, Cd^2+^, Cr^6+^, Ni^2+^ and Zn^2+^ by the same microorganism from galvanoplastic industrial effluents. The results were surprising since the pre-treatment of yeast cell with glucose was more efficient than directly adding glucose to the effluent. Perhaps, the growth achieved with *P. agglomerans* in LB culture medium supplemented with glucose 1% caused the active or inactivated cells of this bacterium to increase their capacity to remove Cd (II).

Abbas *et al*. (2014) described the high capacity of biosorption of heavy metal by biosorbent bacterium (*Staphylococcus saprophyticus*, *Enterobacter cloacae*, *Pseudomonas* sp., *P. aeruginosa, Micrococcus* sp., *Thiobacillus thiooxidans*, *Bacillus* sp., *B. cereus*, *B. licheniformis*, *Geobacillus themodenitrificans*, *Actinomycete* sp, and *Micrococcus* sp).

The data obtained from literature on the influence of pH on the biosorption of cadmium are inconsistent and some studies affirm that the pH could alter the adsorption of metallic ions by biomass, but other studies state that this also varies with the optimal pH values for the adsorbents (biomass), and the type of adsorbents [30-33].

Similar results of cadmium biosorption were obtained by Hou *et al*. [34] who reported cadmium biosorption by *Klebsiella* sp. at pH 5.0 and a temperature of 30°C. In addition, it is important emphasize that the strain of *Klebsiella* sp. has approximately ten times the absorption capacity reported for other strains and is promising for the removal of heavy metals from waste water.

The optimal pH for Cd (II) adsorption for *Saccharomyces cerevisiae* is 6.5 [35] and for filamentous fungi is close to 8.0 [36]. Also, Ozdemir *et al*. [37] using inactivated biomass from *Pantoea* sp. proved the optimal pH for Cd(II) removal is at pH 6.0. Other studies using inactivated cells from different bacterial species such as *Ochrobactrum anthropi* showed optimal removal at pH 8.0 and for *Pseudomonas aeruginosa* PU21, pH 6.0 was also observed as the optimal condition [38, 39].

In our study, the effect of pH over the capacity of Cd (II) absorption shows that living cells as well as inactivated biomass are also able to remove this metal efficiently at low pH values of 3.0. But it was also confirmed that on increasing pH up to 7.0, the sorption availability was even better in the case of the inactivated cells. As mentioned before, the medium pH could also affect the solubility of some ions, and their ionization state will determine their capacity to bind to the carboxylate and phosphate groups present on the bacterial surface, these being powerful carriers of cations [37].

The remaining concentrations of Cd (II) were of 0.016 ppm when *Weissella viridescens* and 0.089 ppm for *Lactobacillus sp* were used [40]. Also, four species of *L. mucosae and L. fermentum* reduced the initial Cd (II) concentration to 0.074 ppm, while two strains of *Streptococcus lactolyticus* and two of *Enterococcus faecalis* showed residual concentrations of 0.651 and 0.633 ppm, respectively and strains of *Pediococcus pentosaceus* showed relatively low biosorption of 0.037 ppm [40, 41]. These data justify once more the biotechnological potential use of *Pantoea agglomerans* as a biosorbent for environments polluted with heavy metals such as Cadmium (II).

Abbas *et al*. [15] described the high capacity of biosorption of heavy metal by biosorbent bacterium (*Staphylococcus saprophyticus*, *Enterobacter cloacae*, *Pseudomonas* sp., *P. aeruginosa, Micrococcus* sp., *Thiobacillus thiooxidans*, *Bacillus* sp., *B. cereus*, *B. licheniformis*, *Geobacillus themodenitrificans*, *Actinomycete* sp, and *Micrococcus* sp).

## CONCLUSION

In the present study, the complete removal of Cadmium Cd (II) by *Pantoea agglomerans* from water by an electrochemical process has been investigated. According to the results from this study, we can affirm that the strain *Pantoea agglomerans* UCP1320 is an efficient biosorbent for Cd (II) in concentrations ranging from 1 to 100 ppm. Living cells or inactivated cells showed optimal removal conditions at 35°C and pH ranging 5.0 to 6.0.

Electrochemical processing is an efficient technology for removing heavy metals from water and this methodology has been attracting increasing interest. This approach of using *P. agglomerans* to remove cadmium represents a promising, simple and cost-effective tool for treating and clearing this heavy metal pollutant from water.

## Figures and Tables

**Fig. (1):**
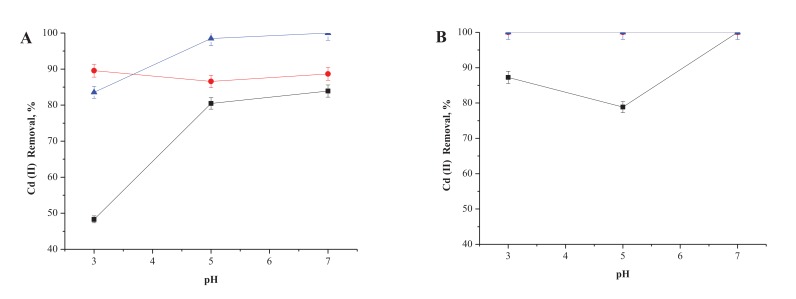
Cadmium adsorption (10 ppm) by inactivated cells of *P. agglomerans* at temperature of 25°C (A) and 35°C (B), respectively on pH 3 to7. Incubation times of 1 h (square), 6h (circle) and 24h (triangle).

**Fig. (2):**
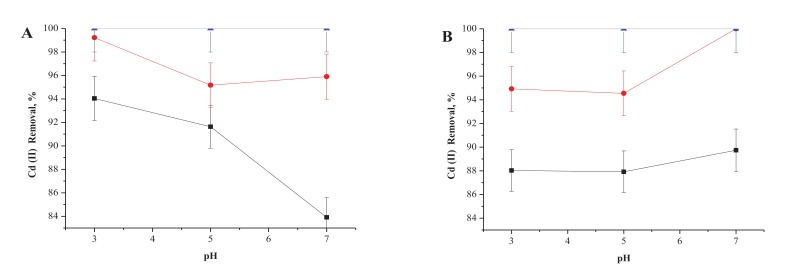
Cadmium adsorption (10 ppm) by living cells of *P. agglomerans* at 25 °C (A) and 35 °C (B), respectively on pH 3 to7. Incubation times of 1 h (square), 6h (circle) and 24h (triangle).

**Fig. (3):**
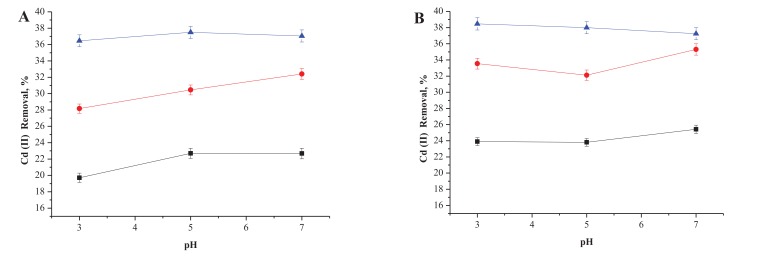
Adsorption of Cadmium (100 ppm) by inactivated cells of *P. agglomerans* at 25 °C (A) and 35 °C (B) , respectively on pH 3 to7. Incubation times of 1 h (square), 6h (circle) and 24h (triangle).

**Fig. (4):**
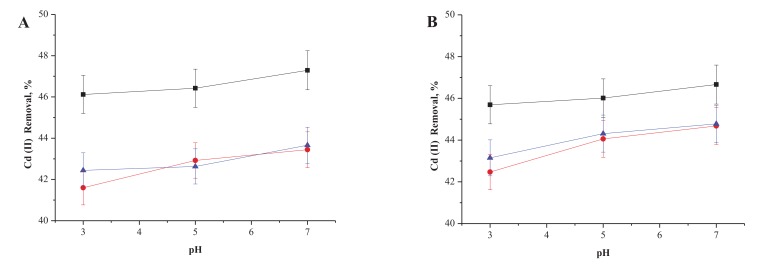
Cadmium adsorption (100 ppm) by living cells of *P. agglomerans* at 25 °C (A) and 35 °C (B) , respectively on pH 3 to7. Incubation times of 1 h (square), 6h (circle) and 24h (triangle).

**Table 1:** Comparison between Cd(II) maximum removals (10ppm) due to the biosorption or biotransformation using inactivated biomass and living cells of *P. agglomerans* incubated for 6h.

**pH/Temperature**	**Both Sorption and Metabolic Removal (%)**		**Only Sorption** **Removal (%)**		**Real Removal (%) Due to Bacterial Metabolism**	
	**25 °C**	**35 °C**	**25 °C**	**35 °C**	**25 °C**	**35 °C**
pH 3.0	99.22	94.92	89.56	87.26	9.66	7.66
pH.5.0	95.17	94.55	86.57	78.85	8.60	15.70
pH 6.0	95.90	100.0	88.66	100.0	7.24	0

**Table 2:** Comparison between Cd(II) maximum removals (at 100 ppm), due to biosorption or biotransformation using inactivated biomass and living cells of *P. agglomerans* incubated for 24h.

**Temperature**	**Both Sorptive and Metabolic Removal (%)**		**Only Sorptive** **Removal (%)**		**Real Removal (%)** **Due to Bacterial Metabolism**	
	**25 °C**	**35 °C**	**25 °C**	**35 °C**	**25 °C**	**35 °C**
pH 3.0	42.44	43.15	36.46	38.47	5.98	4.68
pH.5.0	42.63	44.31	37.49	37.99	5.14	6.32
pH 6.0	43.66	44.78	37.05	37.25	6.61	7.35

**Table 3:** Cd (II) Removal rates (ppm h^-1^)observed for active and inactivated cells of *P. agglomerans* under the conditions studied.

**pH**	**TEMPERATURE**	**Cd (II)**	**INACTIVATED CELLS**			**LIVING CELLS**		
	**°C**	**(ppm)**	**(ppm h^-1^)**			**(ppm h^-1^)**		
3.0	25	10	1.667	±	0.033	5.231	±	0.104
3.0	25	100	4.752	±	0.095	1.483	±	0.029
3.0	35	10	1.609	±	0.032	5.989	±	0.119
3.0	35	100	5.701	±	0.114	6.215	±	0.124
5.0	25	10	1.340	±	0.026	1.463	±	0.029
5.0	25	100	5.067	±	0.101	1.546	±	0.030
5.0	35	10	1.352	±	0.027	6.276	±	0.125
5.0	35	100	5.378	±	0.107	6.550	±	0.131
7.0	25	10	1.525	±	0.030	1.535	±	0.030
7.0	25	100	5.543	±	0.110	1.591	±	0.031
7.0	35	10	1.519	±	0.030	6.324	±	0.126
7.0	35	100	6.032	±	0.120	6.638	±	0.132

## LIST OF ABBREVIATIONS

ASV: = Anodic Stripping Voltammetry
°C: = Degree(s) Celsius
Cd: = Cadmium
ClAg: = Silver chloride
Cr: = Chromium
Cu: = Copper
h: = Hours
H3PO4: = Phosphoric acid
KCl: = Potassium chloride
mg: = Milligrams
min: = Minute
Pb: = Lead
pH: = Potential hydrogen
SWV: = Square Wave Voltammetry
Zn: = Zinc
